# Macular telangiectasia type 2 genetic risk variants associated with clinical characteristics in the Slovenian cohort

**DOI:** 10.1111/aos.70197

**Published:** 2026-07-28

**Authors:** Ajda Kunčič, Nataša Debeljak, Petra Hudler, Janez Smerkolj, Mojca Urbančič

**Affiliations:** ^1^ Faculty of Medicine, Institute of Biochemistry and Molecular Genetics, Medical Centre for Molecular Biology University of Ljubljana Ljubljana Slovenia; ^2^ Faculty of Medicine, Institute of Biochemistry and Molecular Genetics University of Ljubljana Ljubljana Slovenia; ^3^ Faculty of Medicine University of Ljubljana Ljubljana Slovenia; ^4^ Eye Hospital University Medical Centre Ljubljana Ljubljana Slovenia

**Keywords:** area of ellipsoid zone break, genotype–phenotype associations, macular telangiectasia type 2, neovascularization, polygenic risk score, retinal pigment epithelium hyperplasia

## Abstract

**Purpose:**

Early diagnosis of macular telangiectasia type 2 (MacTel) remains challenging, and the contribution of genetic variation to its clinical heterogeneity is unclear. This study investigated associations between MacTel risk variants and clinical characteristics in a Slovenian cohort.

**Methods:**

In this case–control study, including 65 patients with MacTel and 73 healthy controls, participants underwent a complete ophthalmological examination and multimodal imaging. Clinical parameters included best‐corrected visual acuity (BCVA), retinal thickness, area of ellipsoid zone (EZ) break, retinal pigment epithelium (RPE) hyperplasia and neovascularization (NV). Ten MacTel risk variants were genotyped using TaqMan assays. A polygenic risk score (PRS) was calculated, and phenotype–phenotype and genotype–phenotype associations were evaluated using correlation and regression analyses.

**Results:**

Visual acuity declined with progressive retinal changes, including a larger area of EZ break (*ρ* = 0.72), RPE hyperplasia (*ρ* = 0.49) and NV (*ρ* = 0.55; all *p*_adj_ < 0.05). Two variants showed nominal differences in minor allele frequency between groups: rs36259 (*CERS4*) and rs146953046 (*PHGDH*), with the latter associated with higher odds of MacTel (OR = 3.51, *p* = 0.040). The rs11077850‐C allele (*SNHG16*) showed nominal associations with better BCVA (logMAR) (*ρ* = −0.33, *p* = 0.0066), a smaller area of EZ break (*ρ* = −0.27, *p* = 0.032) and lower odds of progressed disease (OR = 0.30, *p* = 0.011). These associations were attenuated after FDR correction. A PRS constructed from five MacTel risk variants, combined with age moderately discriminated patients from controls (AUC = 0.68).

**Conclusion:**

MacTel risk variants are associated with clinically relevant functional and structural retinal changes. Integrating genetic information with multimodal imaging may support risk stratification and earlier recognition in selected patients.

## 1 INTRODUCTION

Macular telangiectasia type 2 (MacTel) is a bilateral, progressive disease characterized by neurodegeneration and vascular changes in the macula. The disease name originates from the characteristic telangiectatic vascular abnormalities observed on fundus examination and fluorescein angiography (Gass & Blodi, 1993; Gass & Oyakawa, 1982). However, advanced imaging techniques have revealed a substantial neurodegenerative component to its pathogenesis. Optical coherence tomography (OCT) demonstrates ellipsoid zone (EZ) disruption and retinal cavities, indicating neurodegeneration (Charbel Issa et al., 2013; Pauleikhoff et al., 2019).

Clinically, MacTel presents with loss of macular transparency, depletion of macular pigment, crystalline deposits, foveal atrophy, retinal pigment epithelium (RPE) hyperplasia, telangiectatic vessels and neovascularization (NV) in advanced stages (Charbel Issa et al., 2013). The disease is restricted to an oval area, termed the MacTel area, centred over the fovea, with a greater horizontal than vertical diameter (Heeren et al., 2020) (Figure 1a). Symptoms usually manifest in the fifth to sixth decades of life, although the age of onset varies considerably. Early symptoms include reading difficulties and metamorphopsia. A gradual decline in visual acuity, accompanied by progressive paracentral scotomas, leads to significant central vision impairment, although legal blindness is rare (Heeren et al., 2020). A recently proposed multimodal imaging‐based classification system incorporates structural alterations relevant to visual function (Chew et al., 2023) (Figure 1b,c). However, certain clinical features may vary among patients, being absent in some and more pronounced in others. Such heterogeneity may reflect underlying genetic variation.

**FIGURE 1:**
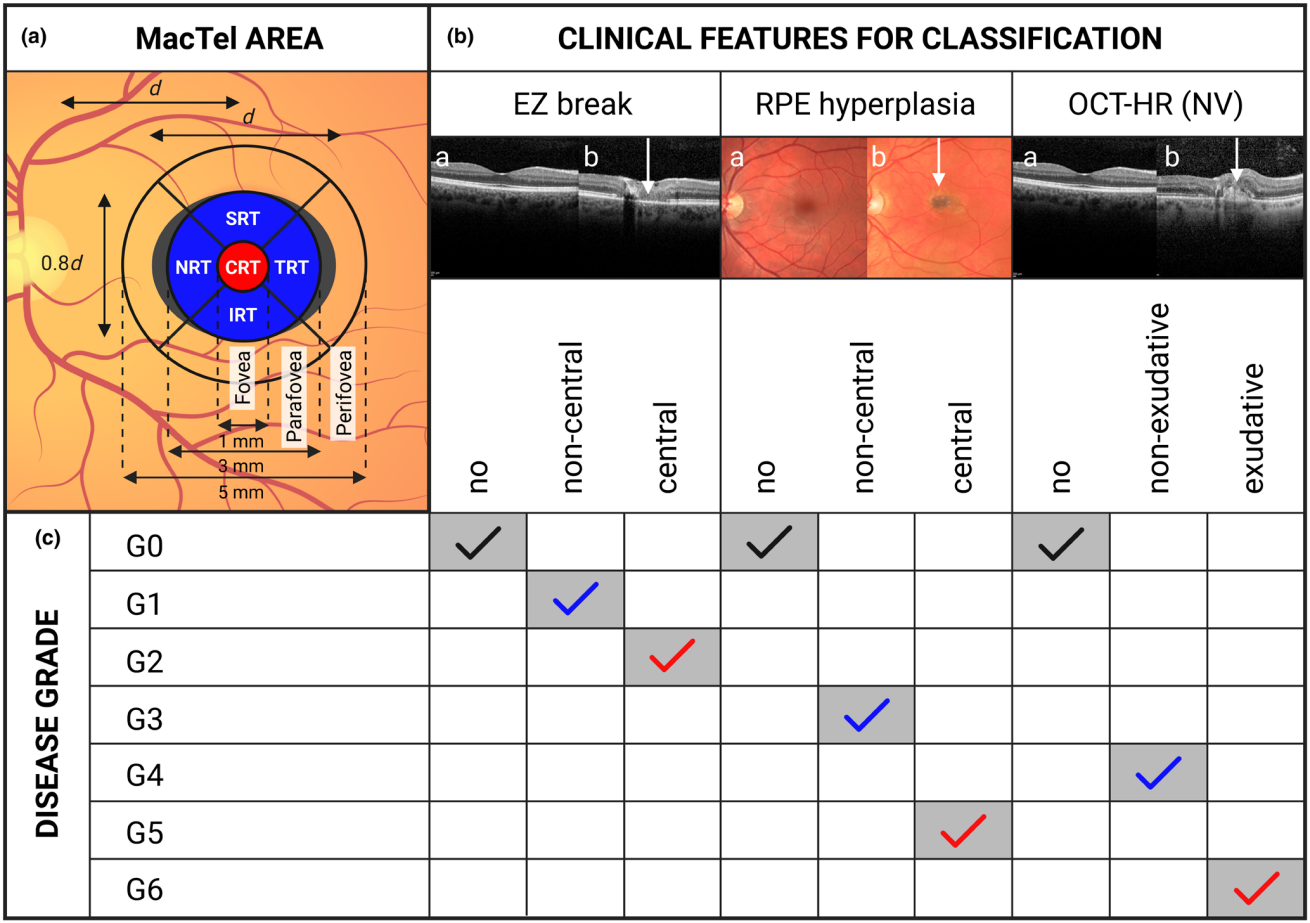
Grading scale for macular telangiectasia type 2 (MacTel). (a) The MacTel area: An oval area (grey oval), centred over the fovea, with a larger horizontal (*d*) than vertical (0.8*d*) diameter (Heeren et al., 2020). The overlaid Early Treatment Diabetic Retinopathy Study (ETDRS) map illustrates the central (CRT) and parafoveal inferior (IRT), nasal (NRT), superior (SRT) and temporal (TRT) subfields encompassing the MacTel area. (b) Three clinical features important for visual function: Ellipsoid zone (EZ) break, retinal pigment epithelium (RPE) hyperplasia and optical coherence tomography hyperreflectivity (OCT‐HR), which reflects intraretinal neovascularization (NV). (a) Healthy fundus image (no clinical features), (b) clinical feature in the CRT subfield indicated by an arrow. (c) Seven disease grades (G0−G6) based on the presence and location of key features defining each grade. Grey boxes with check marks indicate feature location: Not present (black), non‐central (blue) or central (red).

The bilateral presentation, familial occurrence and reports of MacTel in monozygotic twins strongly support a genetic contribution. Early candidate‐gene studies did not find causative variants, indicating a polygenic nature of MacTel (Barbazetto et al., 2008; Parmalee et al., 2010, 2012). Two genome‐wide association studies (GWAS) have since identified eleven significant and eight suggestive loci (Bonelli et al., 2021; Scerri et al., 2017). MacTel risk variants map to genes involved in serine–glycine metabolism (*CPS1*, *GCKR*, *KLF15*, *PHGDH*, *PSPH*, *SLC1A4* and *SLC6A20*), sphingolipid biosynthesis (*CERS4*, *SPTLC1*, *TRIB1AL* and *TTC39B*), lipid processing (*PPP2R5A*, *REEP3* and *SNHG16*) and retinal vascular regulation (*CD109* and *MIR9‐2HG*). The disease likely arises from the combined effects of common variants in metabolic pathway genes, further modified by variants in non‐coding RNAs that modulate gene expression, along with epigenetic and environmental factors. These findings highlight the need to integrate clinical phenotyping with genetic data.

This study aimed to evaluate (1) clinical characteristics of MacTel relevant to visual function, (2) associations between these characteristics and previously identified MacTel risk variants and (3) the effects of key variants identified in the 2021 GWAS in an independent Slovenian cohort. In addition, we present a familial case consistent with possible incomplete penetrance of the examined variants as an illustrative observation.

## 2 METHODS

### 2.1 Participant recruitment

Patients diagnosed with MacTel and managed at the retina clinic at the Eye Hospital, University Medical Centre Ljubljana, Slovenia, were prospectively enrolled in the study. Exclusion criteria were applied per eye: Eyes were excluded if they had additional ocular pathology that could confound the evaluation of clinical characteristics (e.g. age‐related macular degeneration, diabetic macular oedema, macular neovascularization unrelated to MacTel, pathological myopia, central serous chorioretinopathy, epiretinal membrane, significant cataract and corneal or vitreous opacities). Eyes with mild non‐proliferative diabetic retinopathy not involving the macula, mild cataract or pseudophakia were not excluded.

Healthy controls were also prospectively enrolled. Individuals from the general Slovenian population who were referred for examination at the general ophthalmology clinic of the Eye Hospital were screened, and those meeting the criteria were recruited into the study. Inclusion criteria for controls required normal eye status in both eyes. Exclusion criteria were any ocular disease. Controls were frequency‐matched to patients at the group level by age and sex.

Additional exclusion criteria for both groups were refractive error (spherical equivalent) greater than ±2.0 dioptres and neurodegenerative diseases (e.g. multiple sclerosis, Alzheimer's disease, Parkinson's disease). When probands reported a family history of retinal disease, their first‐degree relatives aged 18 years or older were invited for clinical examination. To ensure independence of observations, only unrelated individuals were included in the main case–control analyses.

The study was approved by the National Medical Ethics Committee of the Republic of Slovenia (No. 0120‐112/2023/3, 28 April 2023), and all procedures were in accordance with institutional guidelines and the Declaration of Helsinki. Written informed consent was obtained from all participants prior to enrolment.

### 2.2 Clinical examination

All participants underwent a complete ophthalmological examination, including best‐corrected visual acuity (BCVA) testing (Snellen), anterior segment biomicroscopy and dilated fundus examination, followed by multimodal imaging. Colour fundus photography was acquired using the Optos California ultra‐widefield fundus camera (Optos PLC, Edinburgh, UK). Fundus autofluorescence, infrared‐reflectance fundus imaging, blue‐reflectance fundus imaging and OCT were performed using a Heidelberg Spectralis HRA + OCT machine (Heidelberg Engineering, Heidelberg, Germany). Retinal thickness was automatically measured from OCT images.

For analysis, measurements were extracted from the central (CRT) and parafoveal inferior (IRT), nasal (NRT), superior (SRT) and temporal (TRT) subfields of the Early Treatment Diabetic Retinopathy Study (ETDRS) map, encompassing the MacTel area. The area of EZ break was manually delineated on en face OCT images. The presence of RPE hyperplasia and NV was assessed. Eyes were graded into seven grades (G0 − G6) according to the latest classification system (Chew et al., 2023).

### 2.3 Variants selection

Ten MacTel risk variants were selected for genotyping. Nine common or low‐frequency variants were chosen based on (1) prior association with MacTel as the most significant variant at a genome‐wide significant (*p* < 5 × 10^−8^) or suggestive (*p* < 5 × 10^−6^) locus in the 2021 GWAS (Bonelli et al., 2021) and (2) the availability of TaqMan assays. One rare variant, rs119482081 (*SPTLC1*), known to cause hereditary sensory and autonomic neuropathy type 1 (HSAN1), was additionally included because a subset of patients with HSAN1 carrying this variant has also been reported to develop MacTel.

### 2.4 Genotyping

Peripheral blood was collected into K_2_EDTA tubes (BD Vacutainer^®^, Becton Dickinson, Franklin Lakes, NJ, USA) and stored at −80°C until analysis. Genomic DNA was isolated using the QIAamp DNA Blood Mini Kit (Qiagen, Hilden, Germany) according to the manufacturer's instructions and stored at −20°C until analysis. The concentration and purity of the isolated DNA were determined spectrophotometrically using a Synergy H4 hybrid microplate reader (BioTek Instruments, Inc., Winooski, VT, USA).

Allelic discrimination was performed with TaqMan probes, including *CERS4* rs36259 (C___3098696_40), *CPS1* rs1047891 (C___8793384_1_), *GCKR* rs1260326 (C___2862880_1_), *MIR9‐2HG* rs17421627 (C__33747654_10), *PHGDH* rs146953046 (C__25999264_10), *PPP2R5A* rs2120770 (C__16156980_10), *REEP3* rs10995566 (C___2046176_20), *SLC6A20* rs17279437 (C__25631751_20), *SNHG16* rs11077850 (C___2598751_10) and *SPTLC1* rs119482081 (C__32964504_10) (Applied Biosystems, Thermo Fisher Scientific, Waltham, MA, USA). Genotyping followed the proposed procedure for 5‐μL reactions containing 20× genotyping assay, 2× genotyping master mix, and 1–20 ng of DNA. Polymerase chain reaction (PCR) was performed on a QuantStudio 7 real‐time PCR system using QuantStudio™ real‐time PCR software (both Applied Biosystems, Thermo Fisher Scientific, Waltham, MA, USA). Cycling conditions consisted of a pre‐read stage at 60°C for 30 s, an initial denaturation stage at 95°C for 10 min, followed by a PCR stage of 45 cycles at 95°C for 15 s and at 60°C for 1 min, and a post‐read stage at 60°C for 30 s. All samples and variants were genotyped in duplicate, and concordance between replicates was assessed.

### 2.5 Data analysis and statistics

Data were analysed at the participant or eye levels. For participants with two eligible eyes, values were averaged between the right (RE) and left eye (LE) for participant‐level analyses, whereas both eyes were included in the eye‐level analyses using linear mixed‐effects models with a random intercept for participant to account for within‐subject correlation. For participants with only one eligible eye, data from that eye were used directly. Snellen BCVA was converted to logMAR. Normality was assessed using the Shapiro–Wilk test. Effect sizes were quantified using standardized mean differences (SMD, Hedges' *g*) with 95% confidence intervals (CI).

Demographic characteristics and BCVA (averaged as described above) were analysed at the participant level. Normally distributed continuous variables were compared using Welch's *t*‐test and are presented as mean ± SD with 95% CIs, while non‐normally distributed variables were compared using the Wilcoxon rank‐sum test and are presented as median with interquartile range (IQR). Categorical variables were analysed using the *χ* ^2^ test or Fisher's exact test when expected counts were < 5 and are presented as counts (*n*) and percentages.

Retinal thickness across the central and parafoveal subfields was analysed at the participant level (averaged RE and LE). Mean retinal thickness was calculated as the average of the central and parafoveal subfields. Between‐group differences were evaluated using multivariate analysis of variance (MANOVA). Mean retinal thickness was compared using Welch's *t*‐test, and individual subfields using Welch's *t*‐test with Bonferroni correction. Within‐group differences were assessed using repeated‐measures analysis of covariance (rm‐ANCOVA), with subfield as a within‐participant factor and age as a covariate. The Greenhouse–Geisser correction was applied when sphericity was violated. Age‐adjusted estimated marginal means (EMM) with 95% CIs are reported, and pairwise comparisons were performed using Tukey's honestly significant difference (HSD) test with compact letter display (CLD) notation.

Functional and structural decline across disease grades was analysed at the eye level using mixed‐effects models, with disease grade as a fixed effect and age as a covariate. Age‐adjusted EMMs with 95% CIs are reported, and pairwise comparisons were performed using Tukey's HSD test with CLD notation.

Minor allele frequencies (MAF) were calculated for each variant. Hardy–Weinberg equilibrium (HWE) was evaluated in the control group using an exact test. For each variant, logistic regression was performed with MacTel case–control status as the binary outcome and genotype coded as 0, 1 or 2 copies of the effect allele under an additive genetic model. Associations are reported as odds ratios (OR) with 95% CIs. Multiple testing was controlled using the Benjamini–Hochberg false discovery rate (FDR) method across genetic analyses involving multiple comparisons. Due to the limited sample size, both nominal and FDR‐adjusted *p*‐values are reported.

The polygenic risk score (PRS) was calculated as the weighted sum of effect allele dosages using log(OR) estimates of five genome‐wide significant variants from the 2021 GWAS genotyped in our cohort, including rs1047891 (*CPS1*), rs17421627 (*MIR9‐2HG*), rs146953046 (*PHGDH*), rs10995566 (*REEP3*) and rs17279437 (*SLC6A20*). All variants represent independent loci identified in the 2021 GWAS (Bonelli et al., 2021). PRS values were standardized (*z*‐scores) before analysis. Discriminative ability was evaluated using receiver operating characteristic (ROC) analysis, with the area under the curve (AUC) calculated for PRS alone and PRS combined with age as a covariate, based on logistic regression‐derived predicted probabilities, with 95% CIs estimated using DeLong's method. Logistic regression was used to assess the association between PRS and disease status. For risk stratification, PRS was categorized into tertiles, with the middle tertile as the reference.

Phenotype–phenotype and genotype–phenotype associations were evaluated at the participant level (phenotypic variables averaged between RE and LE) using Spearman's rank correlation coefficient (*ρ*) based on age‐adjusted phenotype residuals.

Associations between genetic risk variants and disease stage were evaluated by comparing eyes with early disease (G0–G2) and eyes with progressed disease (G4–G6; no eyes were graded G3). Mixed‐effects logistic regression under an additive genetic model was used, with age included as a covariate. Results are reported as ORs with 95% CIs.

All statistical analyses were performed in R version 4.4.2.

## 3 RESULTS

### 3.1 Study cohort demographics

In total, 69 patients with MacTel who had already been screened for eye‐level exclusion criteria and 102 controls were recruited. Two invited patients declined participation. Following application of the study selection criteria, two patients and 29 controls were excluded. One patient had Parkinson's disease, and another was excluded to ensure analyses involved only unrelated individuals. All excluded controls were found to have ocular disease during clinical examination. Ultimately, 65 patients with MacTel and 73 controls were included. For five patients, only one eye met the inclusion criteria. The fellow eye was excluded due to ocular pathologies that made reliable assessment of MacTel‐specific clinical features impossible. The reasons for exclusion of the other eye were diabetic macular oedema, status post severe eye trauma, a significant atrophic scar at the posterior pole, status post‐central retinal artery occlusion and choroidal haemangioma. Consequently, 125 eyes (61 RE, 64 LE) from patients and 146 eyes (73 RE, 73 LE) from controls were included in the analyses.

Demographic characteristics and BCVA of the study cohort are summarized in Table 1. Despite a slightly larger control sample, the distribution of age and sex did not differ significantly between groups (*p* = 0.086; *p* = 0.85).

**TABLE 1:** Demographic characteristics and best corrected visual acuity of the study cohort.

Characteristic	Patients	Controls	*p*‐Value
Sex *n* (%)			
Male	34 (52)	37 (51)	
Female	31 (48)	36 (49)	
Total	65 (100)	73 (100)	0.85
Age at study enrolment (years)			
Mean ± SD	67 ± 9	65 ± 8	0.086
Age at diagnosis (years)			
Mean ± SD	64 ± 9	NA	NA
BCVA (logMAR)^a^			
Median [IQR]	0.12 [0.08–0.30]	0.00 [0.00–0.00]	**<0.0001**

*Note*: Bold *p*‐values indicate significant differences (*p* < 0.05).

Abbreviations: BCVA, best‐corrected visual acuity; IQR, interquartile range; *n*, number of subjects; NA, not applicable; SD, standard deviation.

^a^ BCVA (logMAR) at the participant level is reported (averaged right and left eye).

### 3.2 Clinical characteristics

Median BCVA was significantly worse in patients compared with controls (Δ = 0.12 logMAR, 95% CI 0.10–0.20, *p* < 0.0001) (Table 1). Consistent with the asymmetric bilateral presentation of MacTel, median BCVA in patients was 0.15 logMAR [0.10–0.40] in REs and 0.05 logMAR [0.00–0.20] in LEs, whereas controls had 0.00 logMAR [0.00–0.00] in both eyes.

A MANOVA demonstrated a significant group effect on mean retinal thickness (Pillai's trace = 0.50, *F*(5,132) = 25.88, *p* < 0.0001). Patients had reduced mean retinal thickness compared with controls (Δ = −32.1 μm, *p* < 0.0001). Significant thinning was observed across all individual subfields. CRT was reduced by −23.6 μm (*p*_adj_ < 0.0001), while parafoveal reductions ranged from −36.4 μm (TRT) to −32.0 μm (NRT) (all *p*_adj_ < 0.0001) (Table 2).

**TABLE 2:** Retinal thickness (μm) in central and parafoveal subfields: comparison between patients with MacTel and healthy controls.

Subfield	Patients		Controls		Patients vs controls					*p*_adj_‐Value
	Mean ± SD	95% CI	Mean ± SD	95% CI	Δ	95% CI	SMD^a^	95% CI	*p*‐Value	
Mean	295.9 ± 21.3	290.6–301.1	328.0 ± 12.9	325.0–331.0	−32.1	−38.2 to −26.1	1.84	1.44−2.24	**<0.0001**	NA
CRT	257.1 ± 28.4	240.0–264.1	280.7 ± 19.4	276.2–285.2	−23.6	−31.9 to −15.3	0.98	0.62−1.33	**<0.0001**	**<0.0001**
IRT	305.2 ± 21.8	299.8–310.6	339.1 ± 13.8	335.9–342.4	−33.9	−40.2 to −27.7	1.87	1.47−2.27	**<0.0001**	**<0.0001**
NRT	313.7 ± 22.0	308.3–319.2	345.8 ± 13.6	342.6–349.0	−32.0	−38.3 to −25.8	1.77	1.37−2.16	**<0.0001**	**<0.0001**
SRT	308.7 ± 22.2	303.2–314.2	343.3 ± 12.9	340.3–346.3	−34.6	−40.9 to −28.4	1.93	1.52−2.33	**<0.0001**	**<0.0001**
TRT	294.6 ± 26.3	288.1–301.1	331.0 ± 13.6	327.8–334.1	−36.4	−43.6 to −29.2	1.76	1.36−2.15	**<0.0001**	**<0.0001**

*Note*: Bold *p*‐values indicate nominally significant differences (*p* < 0.05) or Bonferroni‐adjusted significant differences (*p*_adj_ < 0.05).

Abbreviations: CI, confidence interval; CRT, central retinal thickness; IRT, inferior retinal thickness; NRT, nasal retinal thickness; SD, standard deviation; SMD, standardized mean difference; SRT, superior retinal thickness; TRT, temporal retinal thickness.

^a^ Standardized mean difference was calculated as Hedges' *g*.

Significant within‐group differences in retinal thickness across the central and parafoveal subfields were observed, even after adjusting for age, in both patients (Greenhouse–Geisser corrected *F*(2.02,127.2) = 200.3, *p* < 0.0001) and controls (Greenhouse–Geisser corrected *F*(1.39,98.6) = 789.5, *p* < 0.0001). In patients, 43% of the retinal thickness variance was explained by subfield (*η* ^2^_G_ = 0.43), compared with 73% in controls (*η* ^2^_G_ = 0.73). Age had a modest effect in patients (*p* = 0.056) but not in controls (*p* = 0.21). In patients, the thickest parafoveal subfield was NRT (313.7 ± 22.0 μm), followed by SRT (308.7 ± 22.2 μm), IRT (305.2 ± 21.8 μm) and TRT (294.6 ± 26.3 μm) (Figure 2a). In controls, NRT (345.8 ± 13.6 μm) and SRT (343.3 ± 12.9 μm) were the thickest parafoveal subfields and did not differ significantly, followed by IRT (339.1 ± 13.8 μm) and TRT (331.0 ± 13.6 μm) (Figure 2b). The largest parafoveal difference was between NRT and TRT, observed in both patients (Δ = −19.2 μm, *p*_adj_ < 0.0001) and controls (Δ = −14.8 μm, *p*_adj_ < 0.0001).

**FIGURE 2:**
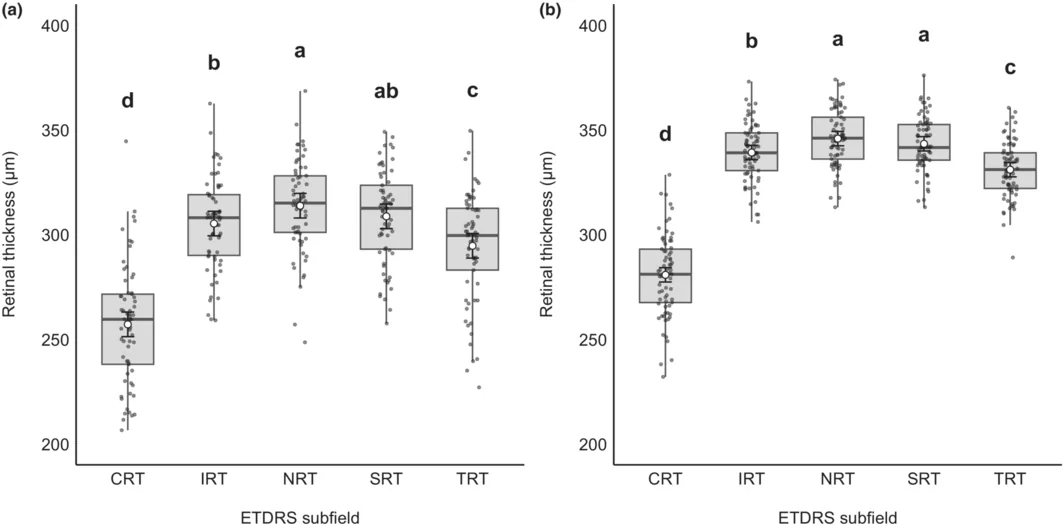
Retinal thickness across central and parafoveal subfields in patients with MacTel (a) and healthy controls (b). Boxplots show retinal thickness (μm) across the central (CRT) and parafoveal inferior (IRT), nasal (NRT), superior (SRT) and temporal (TRT) subfields of the Early Treatment Diabetic Retinopathy Study (ETDRS) map, displaying the median with interquartile range and range (min–max). White circles with bars represent age‐adjusted estimated marginal means with 95% confidence intervals derived from linear mixed‐effects models. Letters above the boxplots indicate results of Tukey‐adjusted pairwise comparisons between subfields. Groups sharing at least one letter do not differ significantly (*p* > 0.05).

EZ break was present in 81% of MacTel eyes (70% bilateral). RPE hyperplasia occurred in 29% of eyes (20% bilateral). Non‐exudative NV was observed in 48% of eyes (32% bilateral) and exudative NV in 10% (7% bilateral). The most frequent disease grade was G4 (27%), followed by G2 (25%), G0 (19%), G6 (11%), G5 (10%) and G1 (7%). No eyes were graded G3. The distribution of clinical features and grades, including bilaterality and total counts, is summarized in Table 3.

**TABLE 3:** Distribution of clinical features and disease grades in MacTel eyes, including bilaterality and total counts.

Parameter	Right eye	Left eye	Bilateral	Total
MacTel eyes *n* (%)	61 (49)	64 (51)	60 (96)	125 (100)
Clinical features *n* (%)				
EZ break	51 (84)	50 (78)	42 (70)	101 (81)
RPE hyperplasia	19 (31)	17 (27)	12 (20)	36 (29)
NV				
Non‐exudative	32 (52)	28 (44)	19 (32)	60 (48)
Exudative	6 (10)	7 (11)	4 (7)	13 (10)
Disease grade *n* (%)				
G0	10 (16)	14 (22)	5 (8)	24 (19)
G1	4 (7)	5 (8)	0 (0)	9 (7)
G2	13 (21)	18 (28)	9 (15)	31 (25)
G3	0 (0)	0 (0)	0 (0)	0 (0)
G4	19 (31)	15 (23)	6 (10)	34 (27)
G5	8 (13)	5 (8)	4 (7)	13 (10)
G6	7 (12)	7 (11)	5 (8)	14 (11)

Abbreviations: EZ, ellipsoid zone; *n*, number of subjects; NV, neovascularization; RPE, retinal pigment epithelium.

The following analyses were performed at the eye level. Visual acuity declined progressively with increasing disease severity, independent of age, which was not significantly associated with BCVA (*p* = 0.27). Eyes with progressed disease (G6) had significantly worse BCVA compared with early stages, indicating a stepwise decline (Figure 3a). The extent of EZ disruption increased progressively with disease severity and was not associated with age (*p* = 0.94). Eyes with progressed disease (G6) exhibited significantly larger areas of EZ break compared with all earlier stages. Differences between early and intermediate stages were not statistically significant, with overlapping distributions observed across G0–G4 (Figure 3b). Mean retinal thickness did not differ significantly across disease grades (*p* = 0.42), while age showed a borderline association (*p* = 0.054). Although a trend towards lower retinal thickness was observed in more advanced stages, differences between grades were small and not statistically significant, with overlapping distributions across all groups (Figure 3c).

**FIGURE 3:**
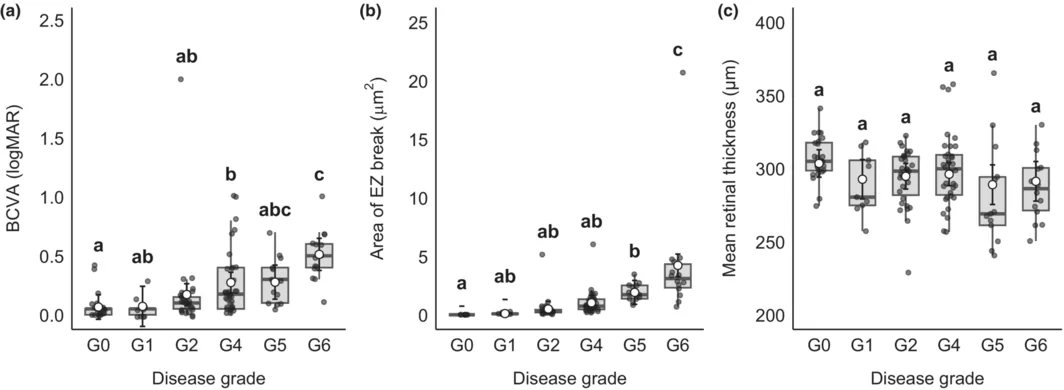
Functional and structural alterations across disease grades (G0–G6). (a) Best‐corrected visual acuity (BCVA; logMAR), (b) area of ellipsoid zone (EZ) break (μm^2^) and (c) mean retinal thickness (μm). Boxplots show the median with interquartile range and range (min–max). White circles with bars represent age‐adjusted estimated marginal means with 95% confidence intervals derived from linear mixed‐effects models. Letters above the boxplots indicate results of Tukey‐adjusted pairwise comparisons between disease grades. Groups sharing at least one letter do not differ significantly (*p* > 0.05). Sample sizes (*n*) for each disease grade are provided in Table 3 and correspond to the total number of eyes analysed.

### 3.3 Genotyping

Concordant genotype calls were obtained for all samples and variants, resulting in a call rate of 100%. No samples or variants were excluded due to poor genotyping quality. All variants with sufficient genotype variability were in HWE (*p* > 0.05). For two variants, rs146953046 (*PHGDH*) and rs17279437 (*SLC6A20*), HWE could not be reliably assessed due to insufficient genotype variability in controls, and rs119482081 (*SPTLC1*) was monomorphic.

Nominal associations with MacTel case–control status were observed for two analysed variants: rs36259 (*CERS4*) (OR = 2.04, 95% CI 1.20–3.47, *p* = 0.0087) and rs146953046 (*PHGDH*) (OR = 3.51, 95% CI 1.06–11.65, *p* = 0.040). The former remained suggestive after FDR correction (*p*_adj_ = 0.078). No participant carried the rare pathogenic rs119482081 (*SPTLC1*) variant (Table 4).

**TABLE 4:** Minor allele frequencies of MacTel risk variants in the Slovenian cohort.

Variant (gene)	Effect allele	MAF patients	MAF controls	OR	95% CI	*p*‐Value	*p*_adj_‐Value^b^
rs36259 (*CERS4*)	G	0.385	0.233	2.04	1.20−3.47	**0.0087**	0.078
rs1047891 (*CPS1*)^a^	A	0.254	0.363	0.59	0.34−1.00	0.052	0.15
rs1260326 (*GCKR*)	C	0.477	0.500	0.92	0.58−1.45	0.71	0.80
rs17421627 (*MIR9‐2HG*)^a^	G	0.115	0.089	1.35	0.61−3.00	0.46	0.60
rs146953046 (*PHGDH*)^a^	G	0.085	0.027	3.51	1.06−11.65	**0.040**	0.15
rs2120770 (*PPP2R5A*)	C	0.177	0.212	0.77	0.40−1.47	0.42	0.60
rs10995566 (*REEP3*)^a^	T	0.262	0.329	0.75	0.46−1.23	0.25	0.46
rs17279437 (*SLC6A20*)^a^	A	0.092	0.048	1.98	0.76−5.16	0.16	0.37
rs11077850 (*SNHG16*)	C	0.208	0.205	1.01	0.55−1.87	0.96	0.96
rs119482081 (*SPTLC1*)	T	0.000	0.000	NA	NA	NA	NA

*Note*: Bold *p*‐values indicate nominally significant differences (*p* < 0.05).

Abbreviations: *CERS4*, ceramide synthase 4; CI, confidence interval; *CPS1*, carbamoyl phosphate synthetase 1; *GCKR*, glucokinase regulator; MAF, minor allele frequency; *MIR9‐2HG*, *MIR9‐2* host gene; NA, not applicable; *PHGDH*, phosphoglycerate dehydrogenase; *PPP2R5A*, protein phosphatase 2 regulatory subunit B'alpha; PRS, polygenic risk score; *REEP3*, receptor expression‐enhancing protein 3; *SLC6A20*, solute carrier family 6 member 20; *SNHG16*, small nucleolar RNA host gene 16; *SPTLC1*, serine palmitoyltransferase long chain base subunit 1.

^a^ Five genome‐wide significant variants included in the PRS calculation.

^b^ Benjamini–Hochberg false discovery rate‐adjusted *p*‐values.

Although only two variants reached nominal significance in the Slovenian cohort, MAF trends were directionally consistent with findings from 2021 GWAS. Higher MAFs in patients were observed for risk variants rs36259 (*CERS4*), rs17421627 (*MIR9‐2HG*), rs146953046 (*PHGDH*), rs17279437 (*SLC6A20*) and rs11077850 (*SNHG16*), whereas lower MAFs for protective variants rs1047891 (*CPS1*), rs1260326 (*GCKR*), rs2120770 (*PPP2R5A*) and rs10995566 (*REEP3*) (Figure S1).

### 3.4 Polygenic risk score

We evaluated the predictive performance of a PRS constructed from five genome‐wide significant MacTel risk variants (Table 4) with age included as a covariate. The PRS was associated with increased odds of MacTel (OR per SD = 1.80, 95% CI 1.22–2.64, *p* = 0.0030) and remained significant after inclusion of age (OR = 1.92, 95% CI 1.29–2.87, *p* = 0.0014). The PRS demonstrated modest discriminatory ability (AUC = 0.66, 95% CI 0.57–0.75), improving slightly after inclusion of age (AUC = 0.68, 95% CI 0.60–0.77). Stratification by PRS tertiles showed a trend towards increasing odds of MacTel with higher PRS. Compared with the middle tertile, individuals in the lowest tertile had lower odds of disease (OR = 0.47, 95% CI 0.21–1.04), whereas those in the highest tertile had higher odds (OR = 2.00, 95% CI 0.81–4.95). However, the CIs were wide and overlapping, indicating that these differences were not statistically significant and reflecting limited precision.

### 3.5 Phenotype–phenotype associations

Associations between clinical characteristics of MacTel were evaluated at the participant level with variables adjusted for age. The complete association matrix is presented in Figure 4. Visual function declined with increasing retinal structural changes. Worse BCVA (logMAR) correlated with a larger area of EZ break (*ρ* = 0.72), presence of RPE hyperplasia (*ρ* = 0.49) and NV (*ρ* = 0.55; all *p* < 0.0001). Reduced mean retinal thickness was associated with larger area of EZ break (*ρ* = −0.38, *p* = 0.0016). These associations remained significant after FDR correction (all *p*_adj_ < 0.05).

**FIGURE 4:**
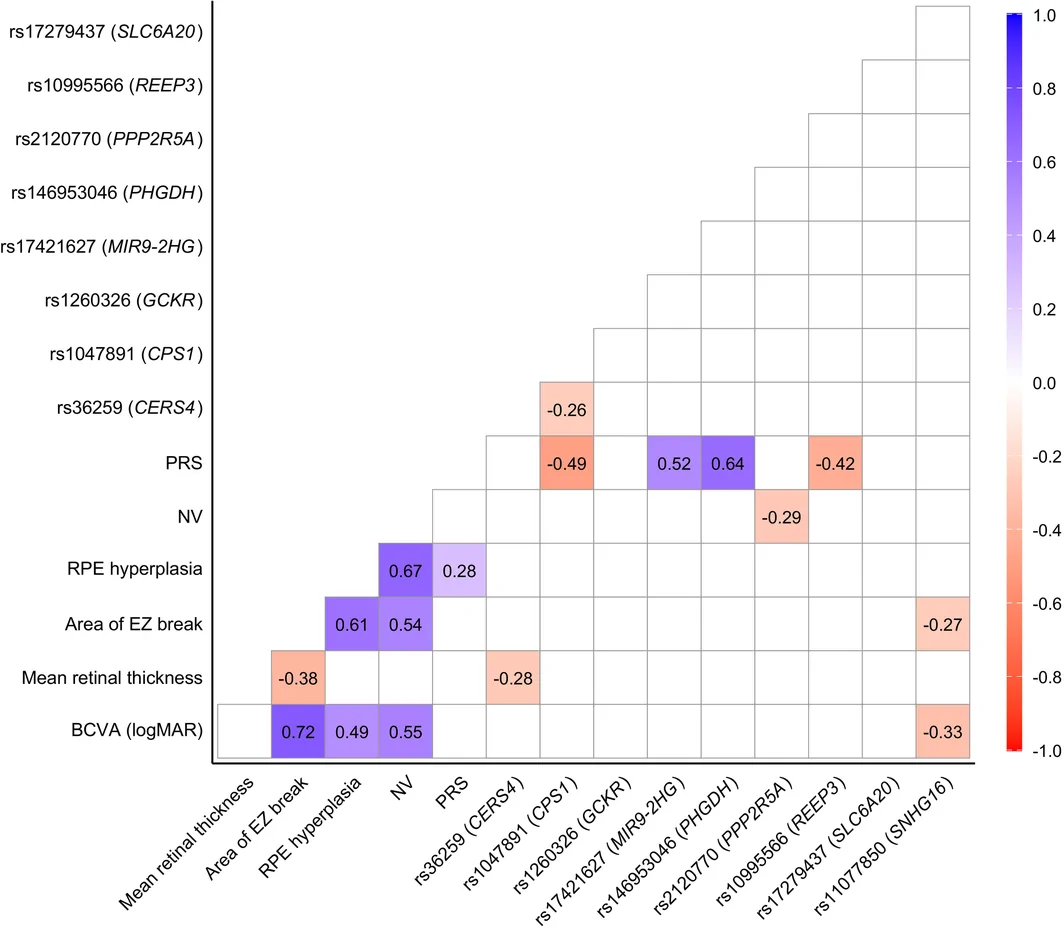
Phenotype–phenotype and genotype–phenotype association matrix. Heatmap showing nominal associations between clinical characteristics of MacTel: Best‐corrected visual acuity (BCVA, logMAR), mean retinal thickness (μm), area of ellipsoid zone (EZ) break (μm^2^), retinal pigment epithelium (RPE) hyperplasia and neovascularization (NV), polygenic risk score (PRS), and selected MacTel risk variants: rs36259 (*CERS4*), rs1047891 (*CPS1*), rs1260326 (*GCKR*), rs17421627 (*MIR9‐2HG*), rs146953046 (*PHGDH*), rs2120770 (*PPP2R5A*), rs10995566 (*REEP3*), rs17279437 (*SLC6A20*) and rs11077850 (*SNHG16*). Phenotypic variables were adjusted for age prior to correlation analysis. Colour represents the direction and strength of associations, with red indicating negative and blue indicating positive associations. Colour intensity reflects the magnitude of the association, categorized as weak (|*ρ*| < 0.3), moderate (|*ρ*| = 0.3–0.5) and strong (|*ρ*| > 0.5). White indicates non‐significant associations (*p* > 0.05).

### 3.6 Genotype–phenotype associations

Among individual variants, weak nominal associations were observed between higher PRS and RPE hyperplasia (*ρ* = 0.28, *p* = 0.024), the rs36259‐G allele (*CERS4*) and lower mean retinal thickness (*ρ* = −0.28, *p* = 0.022), the rs2120770‐C allele (*PPP2R5A*) and absence of NV (*ρ* = −0.29, *p* = 0.017). The rs11077850‐C allele (*SNHG16*) was associated with better BCVA (*ρ* = −0.33, *p* = 0.0066) and smaller area of EZ break (*ρ* = −0.27, *p* = 0.032). The association of the rs11077850‐C allele (*SNHG16*) with better BCVA was suggestive after FDR correction (*p*_adj_ = 0.058), whereas other genotype–phenotype associations were attenuated. The complete association matrix with nominal associations (*p* < 0.05) is presented in Figure 4.

### 3.7 Progression analysis

In the eye‐level progression analysis, eyes with early disease grades (G0–G2) were compared with those with progressed grades (G4–G6). The rs11077850 (*SNHG16*) showed a nominal association with lower odds of progressed disease (OR = 0.30, 95% CI 0.12–0.77, *p* = 0.011) that was attenuated after FDR correction (*p*_adj_ = 0.10).

### 3.8 Familial case report

Among relatives of probands who reported a family history of retinal disease, one family with two affected members (the proband and her mother) was identified. Additional examined relatives included the proband's daughter (29 years old), husband (58 years old) and maternal uncle (68 years old), all of whom showed no clinical characteristics of MacTel (Figure 5). This familial case is presented as an illustrative observation consistent with possible incomplete penetrance of the examined variants, separately from the main case–control study restricted to probands.

**FIGURE 5:**
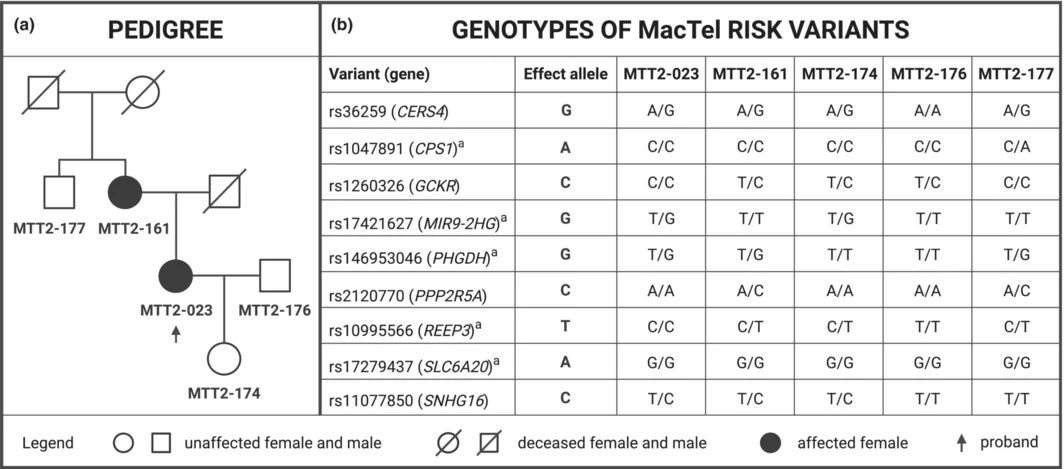
Pedigree (a) and genotypes of MacTel risk variants (b) in a family with two affected individuals. ^a^Five genome‐wide significant variants included in the PRS calculation.

The proband (MTT2‐023), a 51‐year‐old woman, was diagnosed with MacTel four years prior to study enrolment (2024). At enrolment, BCVA was 1.0 logMAR (Snellen 0.05) in the RE and 0.30 logMAR (Snellen 0.5) in the LE, graded as G6 and G1, respectively. She had previously received anti‐VEGF therapy for exudative NV in the RE. Comorbidities included class II obesity (BMI = 32.4), arterial hypertension, asthma and complex regional pain syndrome type 2 following carpal tunnel surgery. Her mother (MTT2‐161), a 75‐year‐old woman, was asymptomatic until diagnosis in 2024. At that time, BCVA was 0.05 logMAR (Snellen 0.9) in the RE and 0.50 logMAR (Snellen 0.3) in the LE, both graded as G2. She also had early age‐related macular degeneration in both eyes and comorbid hyperlipidaemia, arterial hypertension and ulcerative colitis.

Both the proband and her mother carried the non‐protective homozygous genotype C/C for rs1047891 (*CPS1*) and were heterozygous for rs36259 (*CERS4*), rs146953046 (*PHGDH*) and rs11077850 (*SNHG16*). The proband additionally carried the protective homozygous genotype C/C for rs1260326 (*GCKR*), non‐protective homozygous genotypes A/A for rs2120770 (*PPP2R5A*) and C/C for rs10995566 (*REEP3*), as well as a heterozygous genotype for rs17421627 (*MIR9‐2HG*). These differences contributed to her higher PRS (3.09) compared with her affected mother (1.74) and other unaffected relatives, including daughter (0.74), husband (−0.62) and maternal uncle (1.06).

## 4 DISCUSSION

In this study, we evaluated clinical characteristics of MacTel relevant to visual function and assessed their associations with previously identified MacTel risk variants in an independent Slovenian cohort. We calculated a PRS based on five genome‐wide significant variants and described a familial case illustrating possible reduced penetrance of the examined variants, demonstrated by subclinical findings in the asymptomatic mother of a proband with progressed disease.

In our cohort, the mean age at diagnosis and absence of a clear sex predilection are consistent with prior studies (Clemons et al., 2010; Gass & Blodi, 1993). Several eyes graded G0 had symptomatic changes in the fellow eye, and some patients graded G0 in both eyes were identified by chance during routine or unrelated ophthalmic examinations. As previously reported (Heeren et al., 2020), we observed asymmetry between eyes, with a higher proportion of REs graded G4−G5 (44% RE, 31% LE) and more LEs graded G0−G2 (44% RE, 58% LE).

Age‐related retinal thinning is well documented (Nieves‐Moreno et al., 2018; Song et al., 2010; Sung et al., 2009), and previous studies have reported macular thinning in patients with MacTel (Charbel Issa et al., 2008; Krivošić et al., 2009). In our cohort, mean retinal thickness was reduced in patients compared with controls. The lower proportion of variance explained by subfield in patients indicates disruption of the normal spatial organization of retinal thickness due to disease. The modest effect of age observed in patients, but not in controls, suggests age‐related changes consistent with disease progression. Progressive macular thinning is attributed to outer retinal atrophy and correlates with functional impairment (Charbel Issa et al., 2013).

Consistent with the progressive nature of MacTel, multiple morphological alterations occurred simultaneously, with strong associations between area of EZ break, RPE hyperplasia and NV. EZ integrity is a well‐established biomarker of photoreceptor health, and both the extent and proximity of the EZ break to the foveal centre critically determine visual function (Charbel Issa et al., 2007, 2010; Ledolter et al., 2022; Mukherjee et al., 2017). Central involvement and larger EZ breaks are associated with reduced visual acuity and scotomas. Owing to its clinical importance, the new MacTel classification system incorporates EZ break as a key grading parameter (Chew et al., 2023). In our study, EZ breaks were present in 81% of eyes (70% bilateral) and larger EZ breaks strongly correlated with worse BCVA (logMAR). Worsening of visual acuity and enlargement of the EZ break differed significantly across disease grades and were independent of age, whereas differences in retinal thickness showed age dependence.

Most eyes in this study were graded G2 or G4, and none G3. Given the close relationship between grades G3 and G4, this finding is not unexpected. Both grades are closely related, as OCT hyperreflectivity, which is considered to be intraretinal NV, may precede RPE hyperplasia.

Beyond the variants identified in the 2017 GWAS (Scerri et al., 2017), the associations reported in the 2021 GWAS (Bonelli et al., 2021) have not been independently replicated. Although only two of nine examined variants reached nominal significance in the Slovenian cohort, the directionality of MAF differences across all nine variants mirrored GWAS findings (Bonelli et al., 2021). This replication of effect direction, despite the small cohort and limited statistical power, reinforces the central involvement of pathways related to serine–glycine metabolism (*PHGDH*, *CPS1*), sphingolipid synthesis (*CERS4*) and retinal microvasculature regulation (*MIR9‐2HG*) in MacTel pathogenesis.

The missense variant rs36259 (*CERS4*) (p.Ala366Thr) was more frequent in patients (39%) than in controls (23%), consistent with its suggestive association reported in the European cohort of the 2021 GWAS (Bonelli et al., 2021). Ceramide synthase 4 synthesizes very‐long‐chain ceramides (C20 − C26), precursors of sphingolipids. The splice‐donor‐region variant rs146953046 (*PHGDH*) was more frequent in patients (9%) than in controls (3%). The risk haplotype tagged by this variant is associated with reduced *PHGDH* expression in multiple tissues, including the retina (Bonelli et al., 2021). Phosphoglycerate dehydrogenase is the rate‐limiting enzyme for de novo serine biosynthesis. Reduced enzyme activity may produce metabolic stress and contribute to secondary neuroretinal dysfunction (Sinha et al., 2020). Under serine deficiency, serine palmitoyltransferase uses alanine and produces toxic deoxysphingolipids (Gantner et al., 2019; Penno et al., 2010), suggesting a mechanistic link requiring further functional investigation.

The PRS constructed from five genome‐wide significant MacTel risk variants showed modest discriminatory ability between patients and controls (AUC = 0.66), which improved slightly after inclusion of age as a covariate (AUC = 0.68). Similar results were observed when an expanded PRS, including all nine genotyped variants, was evaluated (AUC = 0.68 without age and 0.70 with age), suggesting a limited additional contribution from variants with weaker prior evidence. This performance is lower than that reported for a more comprehensive PRS in 2021 GWAS (Bonelli et al., 2021), which included eleven variants and achieved higher discrimination (AUC = 0.74–0.78 in European cohorts). This difference likely reflects the reduced number of variants included in our PRS, as well as potential differences in allele frequencies and effect sizes in this population. The limited contribution of rs17279437 (*SLC6A20*) in our cohort likely reflects low MAF, a small number of effect allele carriers, highlighting the limited statistical power for low‐frequency variants.

Familial cases remain valuable for understanding the heritable yet variably penetrant nature of MacTel (Gillies et al., 2009; Ronquillo et al., 2018). The proband and her clinically asymptomatic mother both carried multiple MacTel risk variants. The proband had a higher PRS value and was diagnosed at a younger age with more severe disease. This pattern is consistent with possible incomplete penetrance and with contributions from additional genetic and environmental factors. The higher PRS of the proband also supports a dose‐dependent relationship between cumulative genetic risk and clinical phenotype. These findings are consistent with polygenic models of MacTel, in which multiple variants within the serine–glycine–sphingolipid metabolic pathway act additively to modulate disease risk and progression (Bonelli et al., 2021; Eade et al., 2021). This familial case illustrates the continuum from subclinical to progressed disease and underscores the importance of familial screening using multimodal imaging, together with genetic testing, to identify early stage or asymptomatic patients who may benefit from early monitoring and emerging targeted therapies.

Associations between MacTel risk variants and clinical features support the central role of the serine–glycine–sphingolipid axis in MacTel pathogenesis. The PRS captured aspects of cumulative genetic burden, while the familial case illustrates the potential for incomplete penetrance and the value of integrating genetic information with multimodal imaging to support risk stratification and earlier recognition in selected patients. Future studies should combine clinical, genetic, metabolic and environmental factors to develop comprehensive risk‐assessment models.

### 4.1 Study limitations

This study has several limitations. First, the modest sample size, although inherent to a disease such as MacTel, limits statistical power, particularly for low‐frequency variants and genotype–phenotype analyses, and likely contributes to the limited number of significant associations and the wide CIs observed. Second, the PRS was derived from only a subset of genome‐wide significant variants genotyped in our cohort and therefore captures only part of the cumulative genetic risk. Third, the cross‐sectional design precludes assessment of temporal relationships and limits inference regarding disease progression. Consequently, the reported genotype–phenotype associations should be interpreted as exploratory and require confirmation in larger, independent cohorts with longitudinal follow‐up. Nevertheless, generating genetic and phenotypic data in different, including underrepresented, populations remains essential for improving the completeness of our understanding of disease biology.

## FUNDING INFORMATION

This study was supported by the Slovenian Research and Innovation Agency (ARIS), grant number P1‐0390, Junior Researcher funding by ARIS to AK and the University Medical Centre Ljubljana Tertiary Research and Development Project, grant number 20230137.

## Supporting information


**Figure S1.** Comparison of minor allele frequencies (MAF) between the Slovenian cohort and the 2021 GWAS cohort. The figure illustrates directionally consistent MAF trends for all nine MacTel risk variants. Higher MAFs are observed for risk variants rs36259 (*CERS4*), rs17421627 (*MIR9‐2HG*), rs146953046 (*PHGDH*), rs17279437 (*SLC6A20*), rs11077850 (*SNHG16*), whereas lower MAFs for protective variants rs1047891 (*CPS1*), rs1260326 (*GCKR*), rs2120770 (*PPP2R5A*), and rs10995566 (*REEP3*).
