# Supplementary material for: Macular telangiectasia type 2 genetic risk variants associated with clinical characteristics in the Slovenian cohort
Source: Acta Ophthalmol. 2026 Jul 28;104(8):e982–92. doi: 10.1111/aos.70197 (PMC13751882; doi:10.1111/aos.70197)
Supplement: Supplementary file 1 [file AOS-104-e982-s001.pdf]

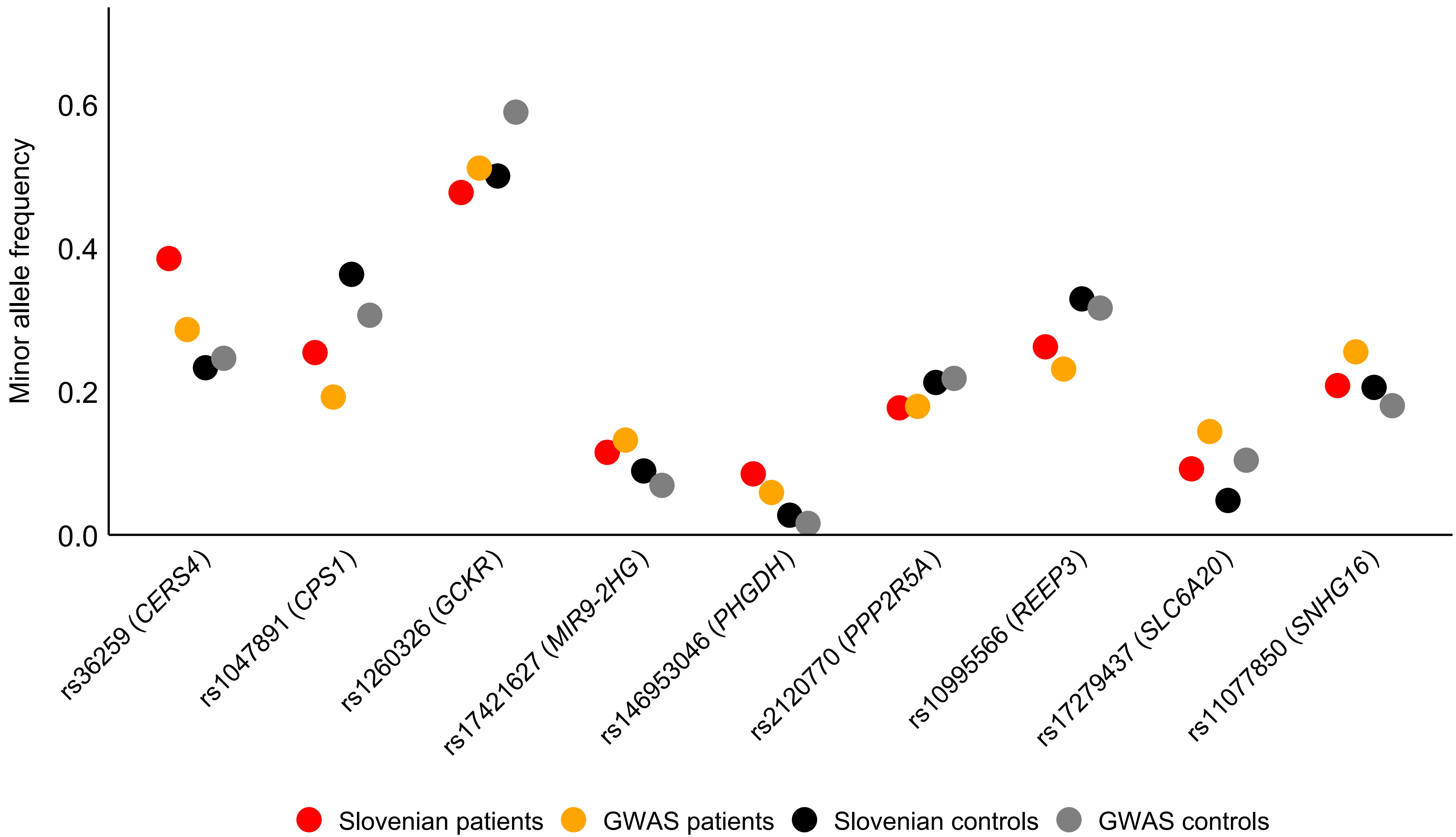
Minor allele frequency
0.0
0.2
0.4
0.6
rs36259 (CERS4)
rs1047891 (CPS1)
rs1260326 (GCKR)
rs17421627 (MIR9-2HG)
rs146953046 (PHGDH)
rs2120770 (PPP2R5A)
rs10995566 (REEP3)
rs17279437 (SLC6A20)
rs11077850 (SNHG16)
Slovenian patients
GWAS patients
Slovenian controls
GWAS controls
